# Supplementary material for: Genome Wide Analysis Points towards Subtype-Specific Diseases in Different Genetic Forms of Amyotrophic Lateral Sclerosis
Source: Int J Mol Sci. 2020 Sep 21;21(18):6938. doi: 10.3390/ijms21186938 (PMC7555318; doi:10.3390/ijms21186938)
Supplement: Supplementary file 1 [file ijms-21-06938-s001.zip › Supplement Figures_revision_20200914-final-clean_BD.docx]

**Supplementary material to manuscript entitled**

“Genome wide analysis points towards subtype specific diseases in different genetic forms of Amyotrophic Lateral Sclerosis”

**Authors**

Banaja P. Dash, Marcel Naumann, Jared Sterneckert, and Andreas Hermann

This file includes:

Supplementary method and figures S1 to S4 with legends

**Supplementary Material - Immunofluorescence staining**

Neurons in culture were washed twice with PBS without Ca2+/Mg2+ (LifeTechnologies) followed by fixation with 4% PFA in PBS for 10 min at room temperature. PFA was discarded and cells were rinsed with PBS another three times. Permeabilization was accomplished by incubation with 0.2% Triton X for 10 min. Afterwards, cells were kept in blocking solution for one hour at RT (1% BSA, 5% donkey serum, 0.3M glycine and 0.02% Triton X in PBS). The primary antibodies were diluted in blocking solution, which was added to the cells and kept overnight at 4 °C. The next day, the primary antibody mixture was aspirated and cells were washed thrice with PBS, which was followed by incubation with the corresponding secondary antibodies also diluted in blocking solution. After another 3 washing steps nuclei counterstaining was performed by addition of Hoechst (LifeTechnologies).

Primary antibodies were used as follows: chicken anti-SMI32 (1:10,000, Covance, PCK-592P), rabbit anti-beta-III-Tubulin (1:3000, Covance, PRB-435P), mouse anti-MAP2 (1:1500, BD Pharmingen, 556320). The secondary antibodies were purchased from Molecular probes (Alexa Fluor 488, Alexa Fluor 555, Alexa Fluor 647) and diluted 1:500.


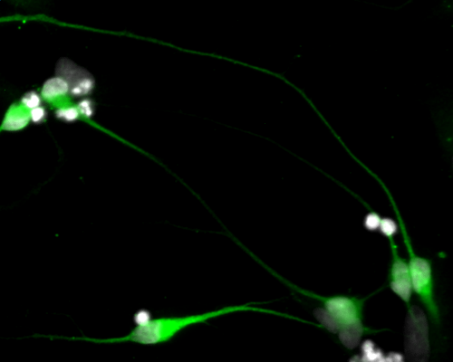

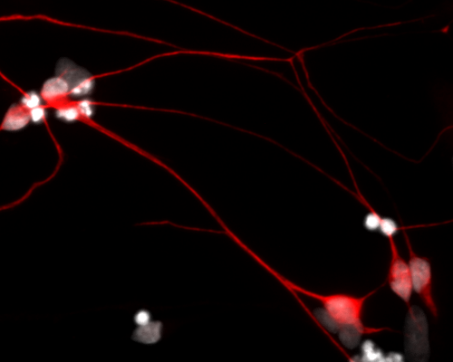

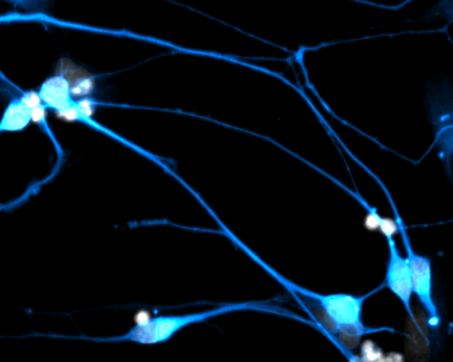

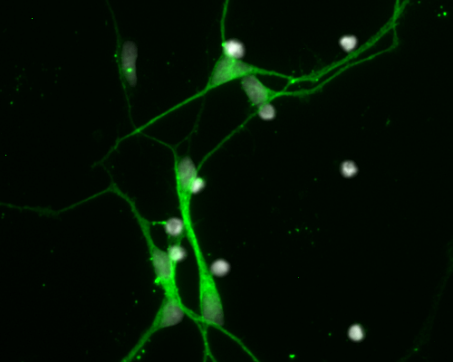

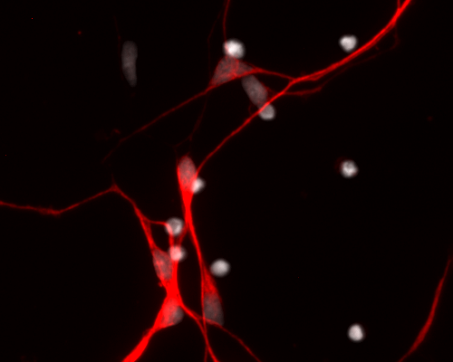

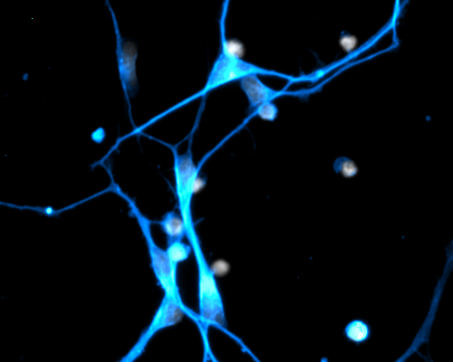

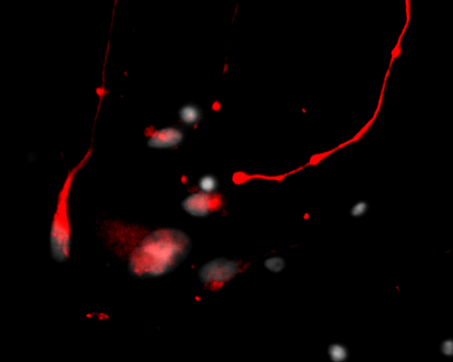

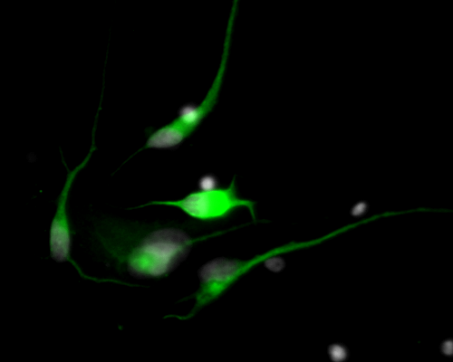

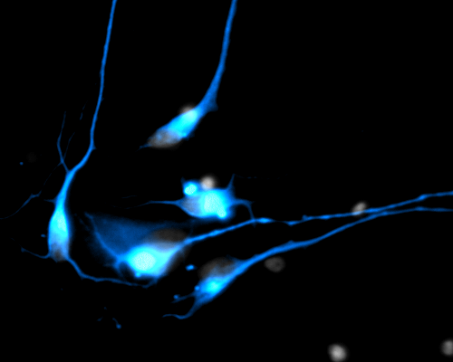


*SOD1* D90A homo

*FUS* R521L hetero

ctrl

SMI32 Hoechst

TUJ1 Hoechst

MAP2 Hoechst

Figure S1: Characterization via immunofluorescence of different neuronal markers in the analysed cell lines. Sufficient neuronal development was seen in healthy controls (WT) and in patient-derived cells with either *FUS* or *SOD1* mutation. Of note is, that the early neuronal differentiation marker TUJ1 (beta-III-tubulin) as well as a mature neuronal marker MAP2 (microtubule-associated protein 2) were observed in parallel with SMI32 (neurofilament heavy chain), which indicates the motoneuron character.


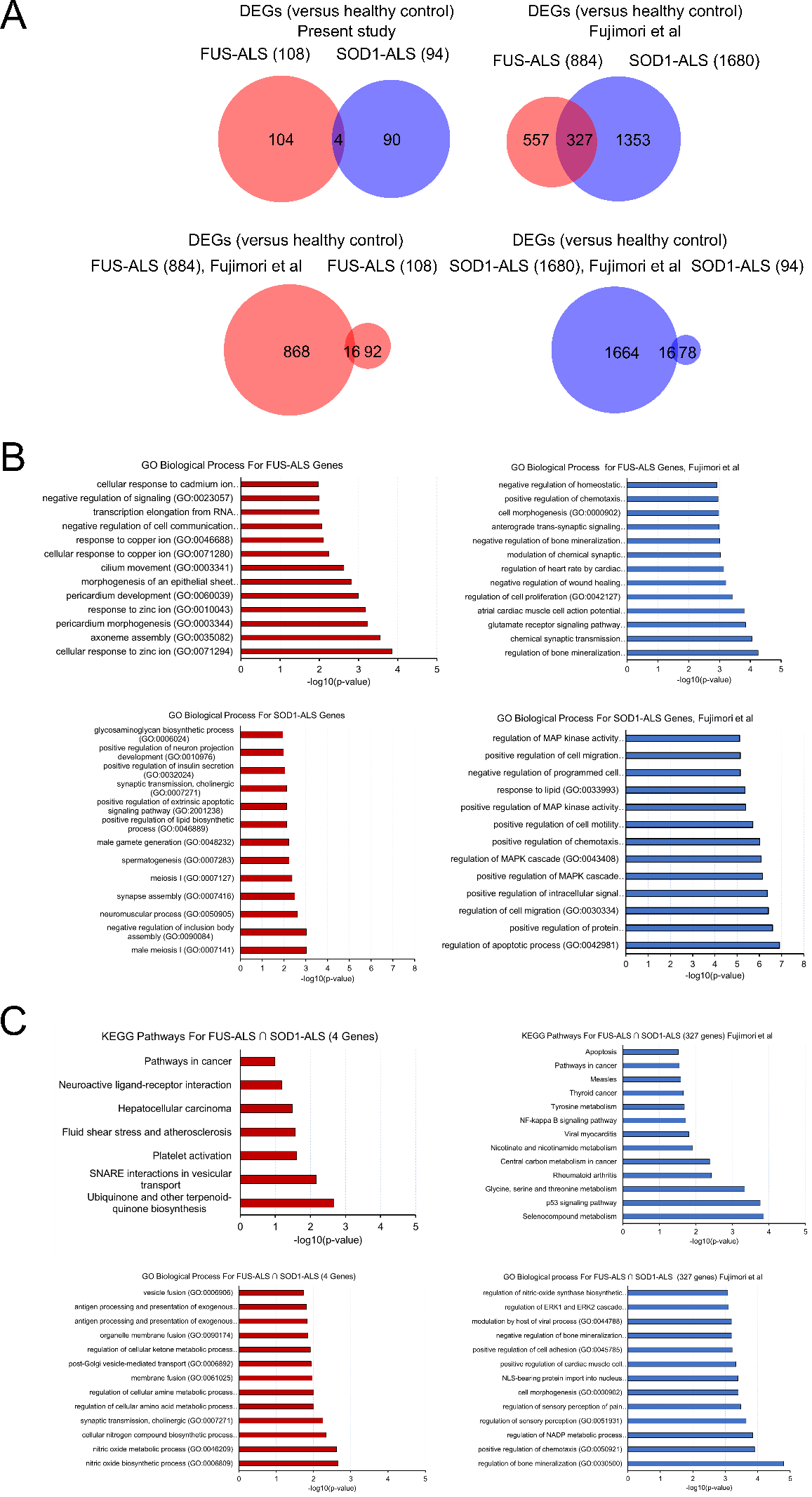


Figure S2: Functional enrichment analysis of DEGs across FUS- and SOD1-ALS datasets. (A) Venn diagrams of commonly expressed and unique DEGs (p-value < 0.05, FC ≥ 0.05) across different combinations of the FUS- and SOD1-ALS (present study (upper left) and GSE106382 (upper right)) and FUS- and FUS-ALS and SOD1- and SOD1-ALS datasets (present study vs GSE106382 (lower left) and present study vs GSE106382 (lower right)). (B) Diagram of GO (Biological Process) terms that are significantly enriched in DEGs. Categories in the upper part of the figure are enriched in DEGs in FUS-ALS datasets (present study andGSE106382). Categories in lower part of the figure are enriched in DEGs in SOD1-ALS (present study and GSE106382). (C) KEGG pathways that were significantly enriched in DEGs. Pathways in the upper part of the figure was performed on the datasets from FUS-ALS DEGs compared to SOD1-ALS DEGs (present study andGSE106382) to identify unique dysregulated pathways whereas the lower part of the figure illustrated significant GO terms (Biological Process) across the same datasets. The statistical significance of the enrichment (-log10(p-value)) was reported for each category.


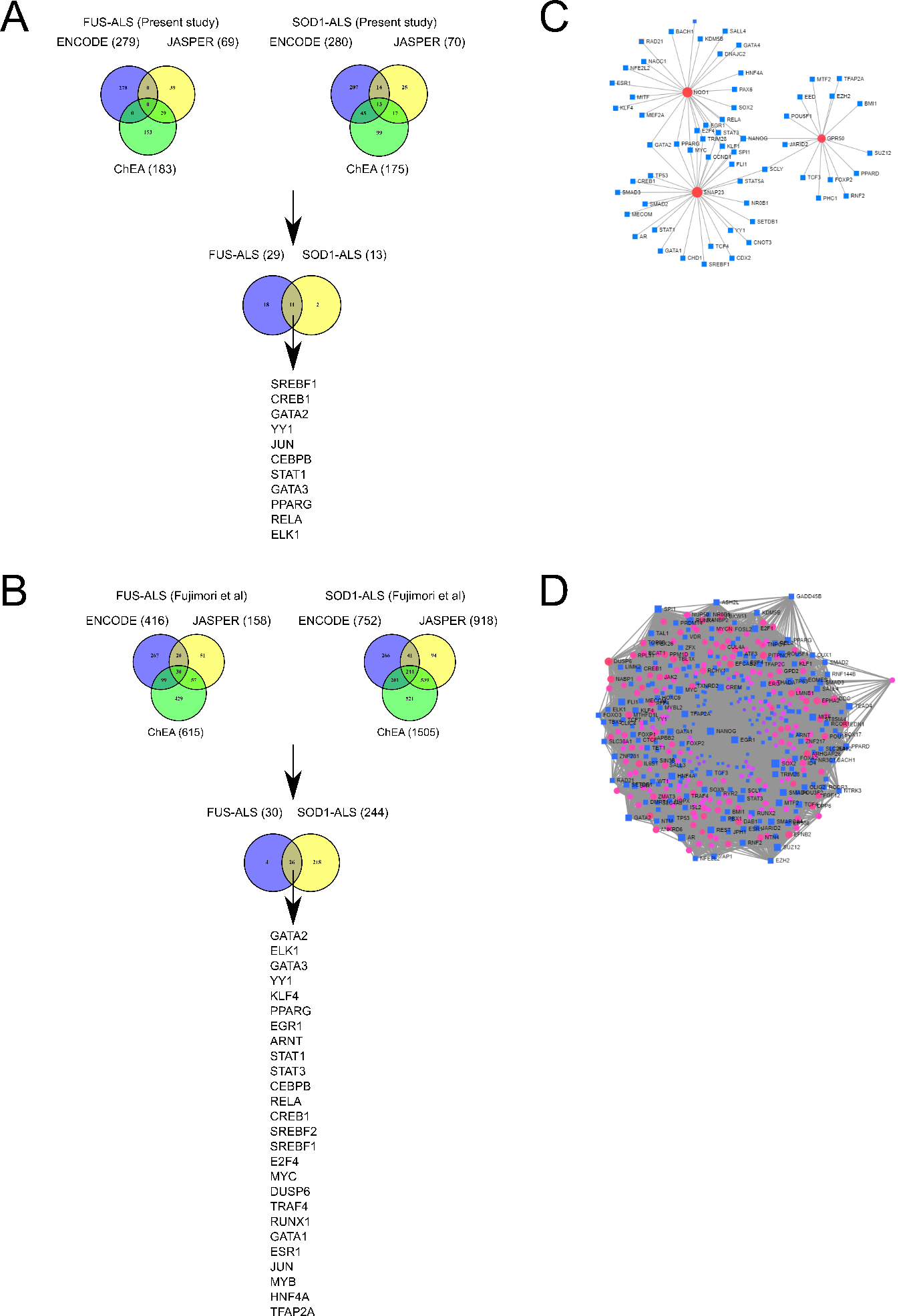


Figure S3: Transcription factor-DEG regulatory interaction network analysis. The DEG-TF interaction network was performed using ENCODE, JASPER and ChEA databases and (A) and (B) represent the results of the Venn diagram analysis constructed with the FUS- and SOD1-ALS genes, (present study and GSE106382) respectively. The common TFs interacting with the FUS and SOD1-ALS DEGs were listed. (C) Construction of regulatory networks of DEG-TF interactions based on DEGs identified shared by FUS- and SOD1-ALS datasets (present study). (D) Construction of regulatory networks of DEG-TF interactions based on a common set of DEGs identified in FUS- and SOD1-ALS datasets (GSE106382) and the minimum connected network was analysed further. All the interaction networks (significance of p < 0.05) were analysed in NetworkAnalyst tool [2]. Venn diagrams were generated using Venny tool [1].


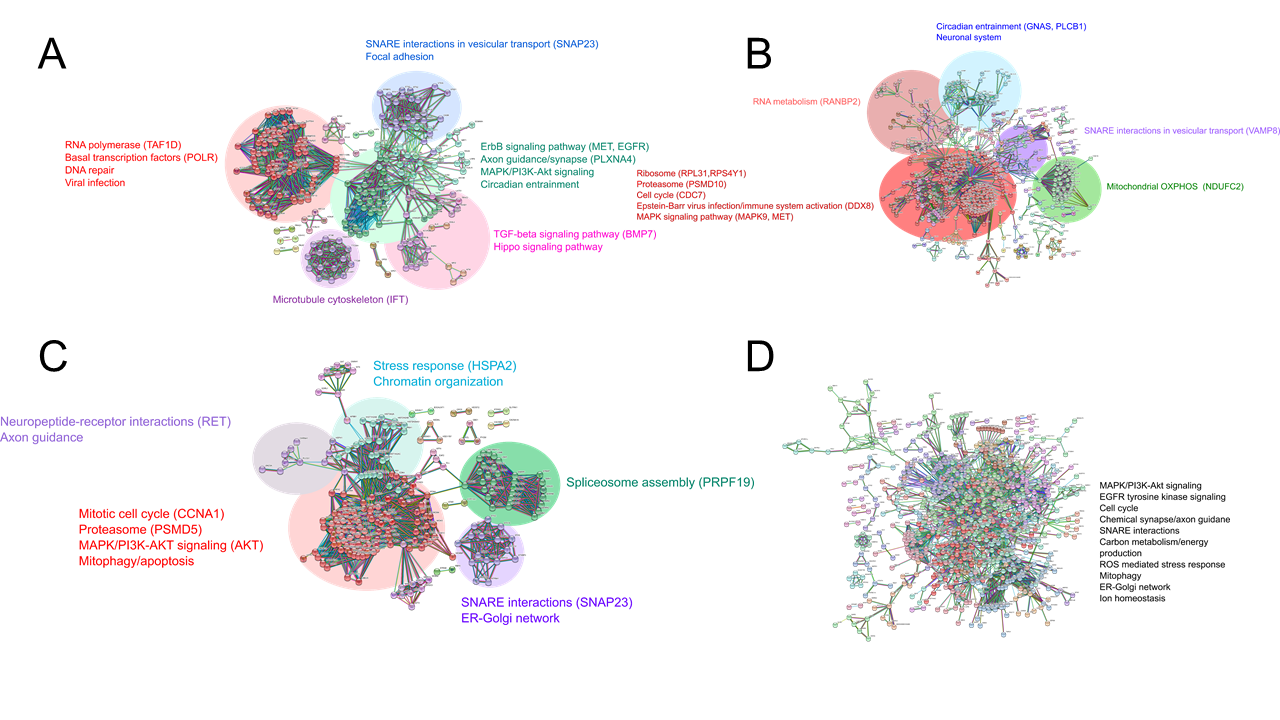


Figure S4: Protein-protein interaction network of dysregulated DEGs in the FUS- and SOD1-ALS datasets (present study, and GSE106382). (A) PPI network was performed on the datasets from FUS-ALS DEGs compared to their controls of this study to identify specific interacting partners and (B) represents PPI network of FUS-ALS DEGs (vs control) with its binding partners unique for GSE106382datasets. (C) PPI network of the DEGs from SOD1-ALS datasets (present study, vs control) along with its unique interacting partners and (D) PPI network of the DEGs with its unique interacting partners in SOD1-ALS datasets (GSE106382 vs control) and the densely connected network was constructed without adding any number of predicted/ known interactions and a Zero-order interaction network was performed to identify most significant hub genes. The nodes indicate the DEGs and the edges indicate the interaction between two proteins. The STRING database [3] was used to establish the interaction network, with highest confidence score of > 0.9 (STRING scores > 0.900) and a maximum number of interactions to top 200 (direct and indirect). MCL clustering (inflation = 1.5) was applied on PPI network to select most significant functional clusters or sub-networks. To identify the most interacting hub genes, we visualized the protein-protein interaction network using NetworkAnalyst tool [2] and analyzed the topological parameters of these nodes (node degree ≥ 15). Clusters of functionally related nodes were manually circled and labelled. Disconnected nodes were omitted. The significant hub genes according to degree and betweenness centrality, with the maximum number of connections, were highlighted in brackets. Statistical significance of p-value < 0.05 was applied in the network.

References:

1. Oliveros, J.C. (2007) Venny. An interactive tool for comparing lists with Venn’s diagrams.https://bioinfogp.cnb.csic.es/tools/venny/index.html.
2. Xia, J.; Gill, E.E.; Hancock, R.E.W. NetworkAnalyst for statistical, visual and network-based meta-analysis of gene expression data. *Nat. Protoc.* **2015**, *10*, 823–844, doi:10.1038/nprot.2015.052.
3. Szklarczyk, D.; Gable, A.L.; Lyon, D.; Junge, A.; Wyder, S.; Huerta-Cepas, J.; Simonovic, M.; Doncheva, N.T.; Morris, J.H.; Bork, P.; et al. STRING v11: Protein-protein association networks with increased coverage, supporting functional discovery in genome-wide experimental datasets. *Nucleic Acids Res.* **2019**, *47*, D607–D613, doi:10.1093/nar/gky1131.
